# Field Exploration for Colony Selection: Evaluating Hygienic Behavior in *Apis cerana indica* Colonies

**DOI:** 10.3390/insects15080598

**Published:** 2024-08-06

**Authors:** Ramkumar Haran, Ettiappan Sumathi, Javaid Iqbal, Sivakumar Krupesh, Ganesan Parthasarathi, Settu Vijay, Vangili Ramasami Saminathan, Madapuji Rajagopalan Srinivasan, Eswaran Kokiladevi, Mannu Jayakanthan, Ali Zeshan

**Affiliations:** 1 Department of Agricultural Entomology, Tamil Nadu Agricultural University, Coimbatore 641003, India; saminathanvr@tnau.ac.in (V.R.S.); mrsrini@tnau.ac.in (M.R.S.); 2 Department of Plant Protection, College of Food and Agriculture Sciences, King Saud University, P.O. Box 2460, Riyadh 11451, Saudi Arabia; jiqbal@ksu.edu.sa; 3 Department of Physical Science and Information Technology, Tamil Nadu Agricultural University, Coimbatore 641003, India; krupesh999sk@gmail.com; 4 Department of Plant Molecular Biology and Bioinformatics, Tamil Nadu Agricultural University, Coimbatore 641003, India; partha1727@gmail.com (G.P.); jayakanthan.m@tnau.ac.in (M.J.); 5 Silkworm Seed Production Centre, National Silkworm Seed Organization, Central Silk Board, Dakshin Bhawanipur, Uttar Dinajpur 733132, India; rsv8016@gmail.com; 6 Department of Plant Biotechnology, Tamil Nadu Agricultural University, Coimbatore 641003, India; kokiladevi@tnau.ac.in; 7 Institute of Agronomic Sciences, Faculty of Agrobiology and Food Resources, Slovak University of Agriculture, 949 76 Nitra, Slovakia; xzeshan@uniag.sk

**Keywords:** *A. cerana indica*, hygienic behavior, pollen, honey, colony metrics, ART-ANOVA

## Abstract

**Simple Summary:**

This study addresses the critical issue of disease resistance in *Apis cerana indica* colonies by focusing on their hygienic behavior and other key characteristics essential for colony health. By evaluating colonies across different seasons and locations in Tamil Nadu, India, this research aims to identify colonies with superior hygienic behavior and overall performance. Through comprehensive field analysis and statistical methods, this study reveals subtle variations, emphasizing the importance of seasonality and location-specific factors. The findings provide valuable insights into the distribution of hive metrics and the relationships between hygienic behavior and other colony parameters. This knowledge will aid in the selection of robust colonies to combat prevailing diseases and pests, enhancing the sustainability and productivity of *A. cerana indica* beekeeping.

**Abstract:**

Hygienic behavior (HB) emerges as a pivotal trait, impacting colony resistance to diseases. This study aimed to understand the behavioral traits of *Apis cerana indica* colonies, with a focus on HB and other key characteristics crucial for colony health, and to screen and identify colonies with superior hygienic behavior and better performance to combat prevailing diseases and pests. This research spans a comprehensive field analysis with different seasons and locations, encompassing the distinct environmental and management factors that influence honey bee behavior. The inclusion of *A. cerana indica* colonies from various locations provides a novel perspective, offering valuable insights regarding the hygienic behavior of *A. cerana indica*. Several statistical analyses, including descriptive statistics, principal component analysis (PCA), and Aligned Rank Transformation-Analysis of Variance (ART-ANOVA) for repeated measures, shed light on the distribution of hive metrics, emphasizing the significance of considering seasonality and location-specific factors. PCA highlights unique characteristics in Tirupur and Coimbatore colonies, while correlation analyses uncover relationships among HB, honey, pollen, brood area, and adult population. Moreover, the study’s nuanced findings gave the status of hygienic behavior of *A. cerana indica* colonies and identified colonies with better colony performance, which will be useful for future breeding programs with *A. cerana indica*.

## 1. Introduction

Honey bees (*Apis cerana*, *A. mellifera*, etc.), functioning as social insects, have successfully established their presence across diverse ecosystems worldwide. They play a crucial role in biodiversity conservation and comprehensive agriculture by serving as essential pollinators not only for crops but also for wild plants [1]. The honey bee colony represents a sophisticated society with a broad spectrum of behaviors designed to safeguard itself from predators and diseases, ensuring reproductive success and overall survival [2,3]. Several of these behaviors hold significance for beekeepers and have consequently been acknowledged in selection and breeding programs [4]. In their natural course, social insects like honey bees, ants, and termites have evolved distinct behavioral adaptations to prevent the spread of pathogens and diseases within their colonies [5,6,7].

Social immunity encompasses traits associated with preventing, reducing, or eradicating pathogens, parasites, and related diseases in social species. Like other eusocial insects, honey bees employ various social immune mechanisms to uphold and enhance the health of their colonies [8]. An inherent aspect of honey bee behavior involves removing infested, diseased, or deceased broods, meticulously cleaning their cells, and transporting these compromised broods out of their hives, a phenomenon termed the hygienic behavior of honey bees [9,10].

Recently, behaviors linked to colony health and disease control, such as hygienic behavior and grooming, have garnered increased attention within colony selection programs [9,10,11,12,13,14,15]. The presence of hygienic behavior in the honey bee species *A. mellifera* was observed and documented in earlier years [16]. This form of social immunity is believed to occur in several social insects [17,18]. Notably, honey bees exhibiting heightened levels of hygienic behavior in their colonies demonstrate greater resistance to two brood diseases, namely American foulbrood [19,20] and Chalkbrood [21]. Honey bees selectively bred for hygienic behavior exhibit elevated levels of this trait, whereas non-selectively bred honey bees display lower levels [22]. Hygienic behavior stands out as one of the most desirable traits in the selective breeding of honey bees by breeders [20].

Beekeepers, particularly those in developing countries, face challenges in recognizing the potential of selecting single multipurpose colonies. Recognizing this need, our study aims to assess the characteristics of honey bee colonies placed in varying conditions when selecting for better performance. Both *Apis mellifera* and *Apis cerana* have been successfully domesticated for pollination services and honey production [23]. While the majority of research has focused on *A. mellifera* and its subspecies, there is a relative paucity of studies examining the behavioral traits of *A. cerana indica*, a species indigenous to South and Southeast Asia.

In southern India, while *A. mellifera* is still present and continues to be introduced, beekeeping with the Indian honey bee *A. cerana indica* is more prevalent. *A. cerana indica* is the only native *Apis* species that has been domesticated in India. However, it remains challenging to reliably differentiate between wild and managed *A. cerana* [24]. *A. cerana indica*, commonly known as the Indian honey bee, is of significant interest due to its indigenous nature and its role in the pollination of a wide range of crops [25,26]. Moreover, *A. cerana indica* is resistant to several pests and diseases that severely affect *A. mellifera* colonies, such as the *Varroa destructor* mite [27]. This resistance is partly attributed to its effective grooming and hygienic behaviors, which play a crucial role in maintaining colony health [28]. These behaviors are integral to the social immunity of *A. cerana indica* and contribute to its resilience in the face of environmental stressors and pathogen challenges.

However, the Thai sac brood virus (TSBV) disease posed a major threat to the hives of *A. cerana indica*, which killed about 95% of *A. cerana* colonies in India [29,30]. As a viral disease, there is no cure because viruses integrate into the host cells [31]. Despite numerous strategies, the disease remains unmanageable. Therefore, the most effective control method seems to be either collecting colonies that have survived the disease or selecting and developing a resistant strain [30].

With this background focusing on *A. cerana indica* colonies from various locations, a preliminary field analysis was conducted to evaluate the key characteristics of these honey bee colonies. Our study extensively investigated hygienic behavior and colony growth parameters, exploring the multiple traits among colonies in two different seasons. These insights aid in identifying robust and profitable colonies for selective breeding while also considering the seasonal implications on the traits evaluated.

## 2. Materials and Methods

### 2.1. Experimental Site

This research employed a total of 286 colonies from beekeepers across diverse locations encompassing six districts (Coimbatore (n = 24), Dindigul (n = 56), Erode (n = 49), Kanyakumari (n = 68), Madurai (n = 52), and Tirupur (n = 37)) within the Tamil Nadu state, India. The details of the locations are provided in Table S1. These locations are representative of the populations of *A. cerana indica,* which are considered to vary due to geographic isolation. The study utilized honey bee hives following the Marthandam design, each equipped with 6 frames of combs for preliminary screening. The colonies showing more than 60% hygienic behavior were considered for further observation and analysis. The experiments were conducted during the honey flow season (February 2023) and the dearth period (October 2023) with the same colonies, and no replacements were made. All colonies featured a fertile queen (age is not recorded), worker bees, broods, honey, and pollen. Notably, no indications of disease were observed in any of the colonies.

### 2.2. Pin-Killed Brood Assay

We performed the pin-killed brood assay to evaluate the hygienic condition of each colony [12,32,33]. Sealed brood combs were selected, and a section of the brood area containing 25 cells was marked. The brood present in the marked area was killed with a sterile entomological pin. After 24 h, the number of dead broods removed by honey bees was recorded. Hygienic behavior % (HB%) was calculated using the following formula.

(1) $$HB\%=\frac{No.\;of\;brood\;removed}{Total\;no.\;of\;broods\;pin-killed}\times 100$$

The colonies showing more than 60% hygienic behavior were selected from initially evaluated colonies and were subject to colony growth parameters (117 out of 286 colonies). The experiments were replicated thrice in 15-day intervals in both seasons for the initially screened 117 colonies (Coimbatore (n = 10/24), Dindigul (n = 21/56), Erode (n = 22/49), Kanyakumari (n = 29/68), Madurai (n = 23/52), and Tirupur (n = 12/37)).

### 2.3. Colony Growth Parameters

The colony growth parameters, namely brood, pollen, and honey area in the comb, were recorded [34]. The assessment of the sealed brood area (BA) involves the placement of a transparent sheet featuring premarked 1 cm^2^ grids on the brood comb. The total brood area was determined by visually inspecting and summing the areas covered by the sealed brood. Concurrently, the enumeration of adult bee populations was conducted using a transparent sheet with grid markings, mirroring the method employed for sealed brood area measurement. The adult bee population (AP) was computed for each 1 cm^2^ grid. Similar to the sealed brood area estimation, honey (HA) and pollen storage (PA) evaluation utilized a transparent sheet with grid marks. The HA, PA, and BA are expressed in cm^2^, whereas the AP is expressed in the number of adults per frame. Initially, the parameters were set as equal in all colonies (three frames in a colony with ~20 cm^2^ HA per frame, ~20 cm^2^ PA per frame, ~30 cm^2^ BA per frame, and approximately 1000 adults per frame) prior to observation. The observations of the colony growth parameters were taken 1 month after equalizing the parameters. This experimental procedure was replicated on three frames per colony, treating each frame as an independent replicate. The experiments were replicated thrice in 15-day intervals in both seasons for the initially screened 117 colonies.

### 2.4. Statistical Analysis

Data were characterized using descriptive statistics. Pearson’s correlation was used to evaluate the relationship between all the traits of interest. To explore the underlying structure and patterns of the data, Principal Component Analysis (PCA) was performed on the collected variables. The phenotypic data obtained as a mean of two seasons was subjected to cluster analysis to group the colonies.

A two-way Analysis of Variance (ANOVA) for repeated measures was conducted, with season and location as the fixed factors and each of the characters as the dependent variable. However, preliminary analyses revealed that the data did not meet the assumptions of normality and homogeneity of variance required for an ANOVA, even after applying various transformations. Hence, to analyze the data, the Aligned Rank Transformation (ART)-ANOVA, a non-parametric alternative, was employed. This method aligns and ranks data values within each factor level before applying ANOVA. ART-ANOVA allows the testing of the main and interaction effects using the F-statistic and *p*-value and also estimates the effect size with partial $\eta_{p}^{2}$. The post-hoc tests were performed to examine the pairwise comparisons of the adjusted means of the groups by the use of contrast analysis. For each contrast, Cohen’s d statistic was calculated to estimate the effect size of each contrast corrected for a small sample size, when necessary, which indicates the standardized mean difference between the groups.

For colony selection with multiple traits, we employed two methods: (a) Z score-based selection index calculation and (b) Hierarchical agglomerative clustering (HAC), considering all the parameters observed in the colonies.

A selection index was employed to select colonies for multiple traits, as suggested by Rinderer (1986) [35]. The selection index was calculated by summing the Z scores for each of the parameters studied. The colonies were then ranked in descending order based on their total Z scores [34]. The Z scores for each parameter were calculated using the formula:

Z_x_ = [(x − a_x_)/a_x_] × 100 (2)

where x represents the value of the parameter for an individual colony and a_x_ is the average value of that parameter across all colonies.

Hierarchical agglomerative clustering (HAC) was performed to group and select the best-performing colonies. HAC was performed with each trait standardized to ensure an equal contribution to the clustering process. Pairwise distances between colonies were calculated using Euclidean distance. Ward’s method, which minimizes total within-cluster variance, was utilized. The dendrogram was segmented into 5 clusters based on k means clustering. All statistical analyses were performed using the R software package version 4.3.1.

## 3. Result

### 3.1. Descriptive Statistics

**Figure 1:**
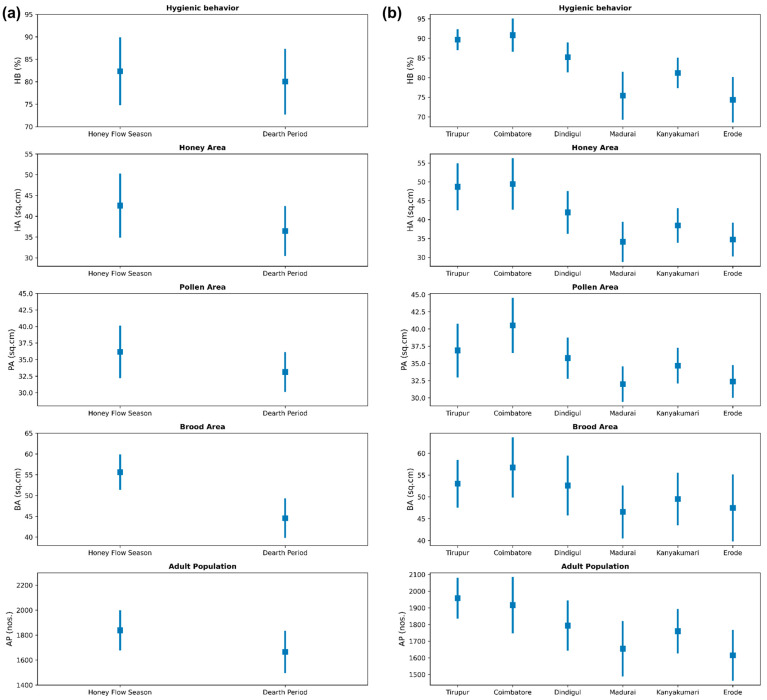
Descriptive statistics of colony traits in (**a**) different seasons and (**b**) different locations.

The results of the descriptive statistics for honey bee colonies across seasons (honey flow season and dearth period) (Figure 1a) and different locations (Figure 1b) revealed intriguing insights into the dynamics of hive metrics. In the honey flow season, the mean HB stood at 82.35 ± 7.39%, while the HA and PA displayed means of 42.59 ± 7.51 cm^2^ and 36.17 ± 3.82 cm^2^, respectively. The BA exhibited a mean of 55.62 ± 4.05 cm^2^, and the AP showcased a mean of 1838.79 ± 154.12 adults. Transitioning to the dearth period, the HB had a mean of 80.03 ± 7.12%, while the HA and pollen area PA recorded means of 36.47 ± 5.76 cm^2^ and 33.14 ± 2.87 cm^2^, respectively. The BA displayed a mean of 44.57 ± 4.52 cm^2^, and the AP presented a mean of 1665.32 ± 163.29 adults. These results illuminate subtle variations in hive metrics between the two seasons, with the honey flow season tending towards marginally higher mean values. On consideration of location, the results revealed that Coimbatore exhibited the highest average hygienic behavior (90.85 ± 3.67%) and high values for brood area (56.75 ± 2.33 cm^2^), with an adult population of 1916.77 ± 110.70. Tirupur followed closely with an average hygienic behavior of 89.71 ± 1.91% and the highest adult population (1958.22 ± 53.94). In contrast, Erode and Madurai displayed the lowest hygienic behavior averages (74.37 ± 5.53% and 75.39 ± 5.72%, respectively) and had lower values for other parameters, indicating poorer hive health. These highlights underscore the variability in hive performance, with Coimbatore and Tirupur leading in most metrics. The detailed descriptive statistics of each season and location are provided in Tables S2 and S3, respectively. To further study the season and location effects, an ANOVA was performed.

### 3.2. Principal Component Analysis

**Figure 2:**
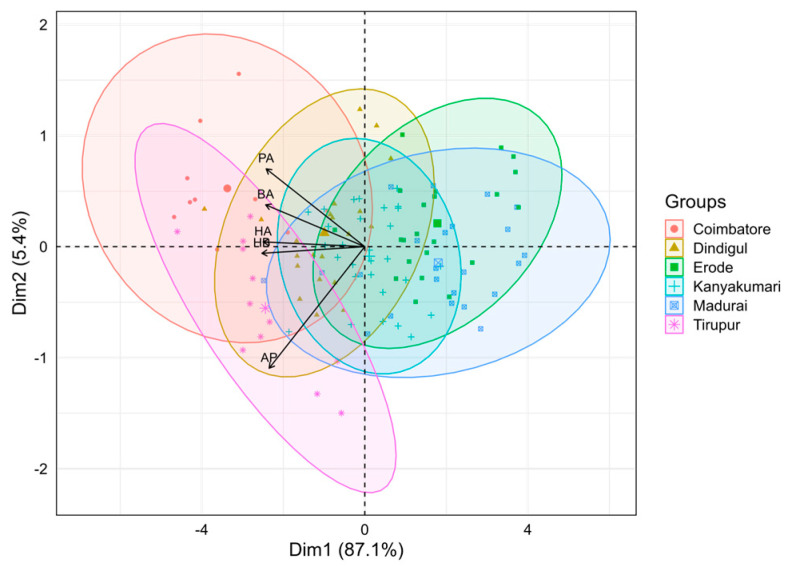
PCA biplot showing colony clusters with traits as vectors.

Principal Component Analysis was performed to understand the distribution and distinctiveness of locations concerning the selected characteristics of honey bee colonies for study (Figure 2). The first two PCs accounted for 92.5% of the total variability among the variables investigated. Traits such as the PA, BA, and HA had positive loadings on the PC2 and exhibited positive correlations with each other. Similarly, the traits HB and AP had negative loadings on the PC1 component and exhibited negative correlations with traits PA, BA, and HA. Groups such as Kanyakumari, Madurai, and Erode were skewed towards the positive quadrant and exhibited greater influence in the PC1. However, Coimbatore, Tirupur, and Dindigul accounted for much variability in the PC2. From the analysis, it was observed that Tirupur and Coimbatore colonies were distinctly distributed and placed distant from the origin, which implies that these groups had significant variations explained by the original variables. Vectors represent the component loadings of the original variables. All five vectors (HB, HA, PA, BA, and AP) were almost equal in length, denoting their more or less equal contribution in explaining the variance. From the direction of the vectors, it was understood that Tirupur colonies could be characterized by higher values of AP, HB, HA, and PA, while HB, HA, BA, and PA were strongly associated with Coimbatore. As far as other locations are concerned, variation among the groups seemed to be less, as huge significant overlaps were found. Particularly, Dindigul and Kanyakumari colonies were clustered around the origin, showing very low variance among the original variables.

### 3.3. Correlation Analysis

**Figure 3:**
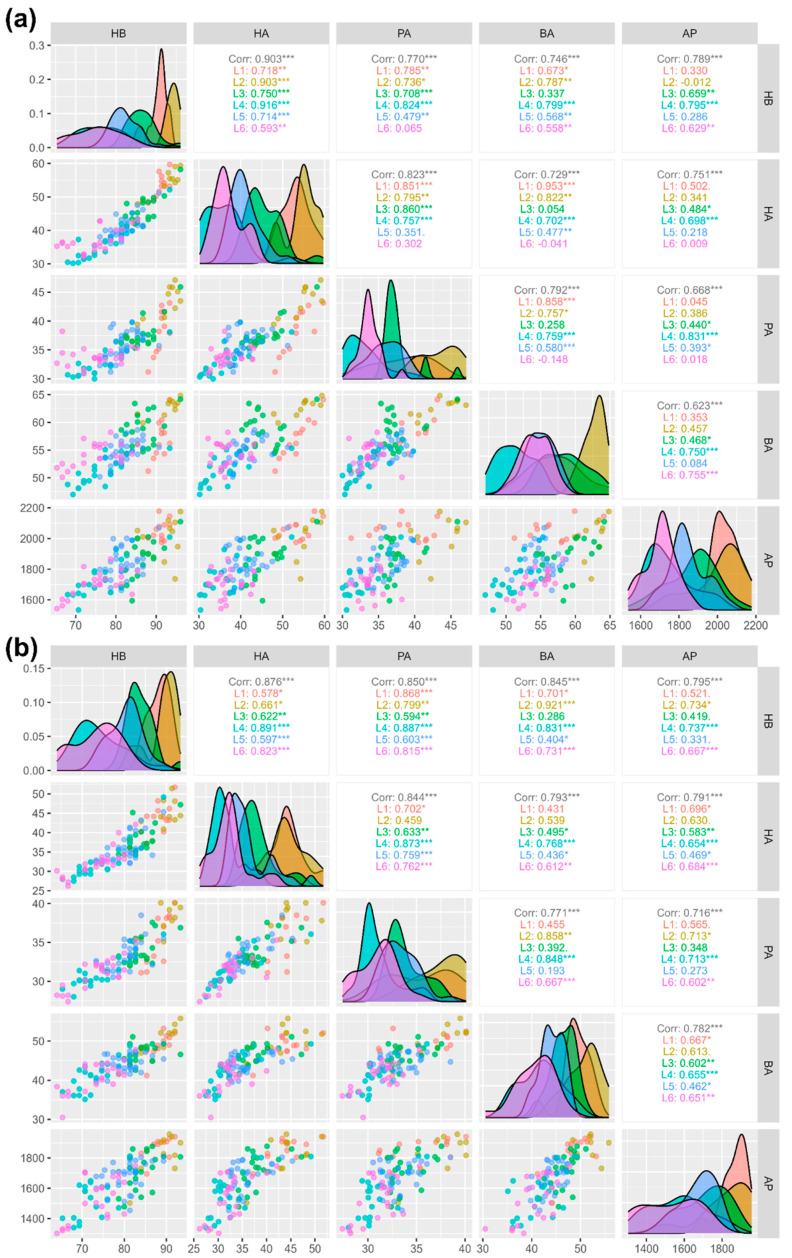
Correlation analysis of colony traits (**a**) honey flow season and (**b**) dearth period. Note: L1—Tirupur, L2—Coimbatore, L3—Dindigul, L4—Madurai, L5—Kanyakumari, and L6—Erode. The significance levels are represented by ‘*’ (*p* < 0.05), ‘**’ (*p* < 0.01) and ‘***’ (*p* < 0.001).

Upon comparing the correlation coefficients across two seasons for the five variables, distinct patterns emerge (Figure 3a,b and Table S4). Hygiene behavior (HB) showed strong positive correlations with both honey area (HA) and adult population (AP), indicating that higher HB values corresponded to elevated HA and AP values. The HA had a substantial positive correlation with the pollen area (PA), while its correlations with the brood area (BA) and AP were comparatively weaker. The PA displayed moderate to strong positive correlations with all other variables, except for a relatively weaker but still significant correlation with the BA. The BA showed weak to moderate positive correlations with all other variables, and AP demonstrated moderate correlations with the remaining variables, except for a strong correlation with HB. These patterns revealed the interdependencies among the variables across the two seasons. Notably, the HB of the colonies had a weaker correlation with the AP in all locations except for Madurai. In Dindigul, the HA and PA showed no significant correlation with the BA, while in Madurai, the adult population had significantly higher correlations with all other variables compared to other districts. In Kanyakumari and Erode, the correlation patterns among the variables were similar.

### 3.4. Analysis of Variance

**Figure 4:**
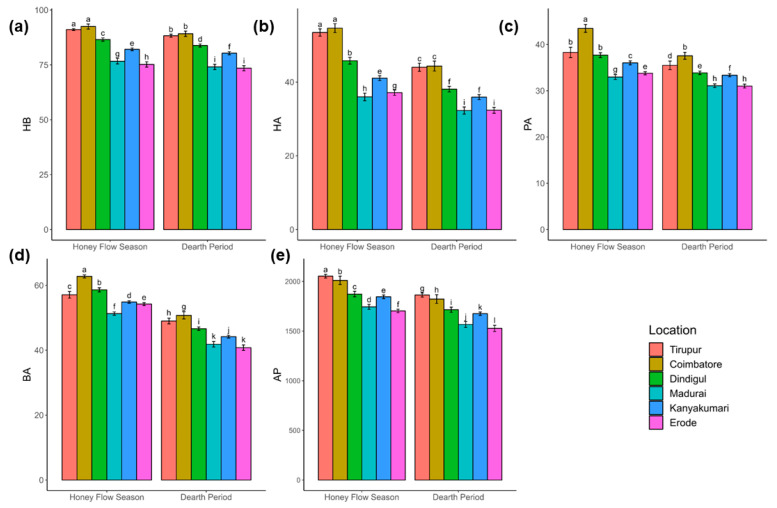
Pairwise mean comparison among characters. The figure shows the average levels of five characters, (**a**) hygienic behavior, (**b**) honey area, (**c**) pollen area, (**d**) brood area, and (**e**) adult population, measured from six locations (Tirupur, Coimbatore, Dindigul, Madurai, Kanyakumari, and Erode) during two distinct periods (honey flow season and dearth period) using bar graphs. The average Cohen’s d effect size was used to quantify the difference between groups and is represented by alphabetic letters. Groups with the same alphabetic letter indicate that differences between those groups are not significant.

**Figure 5:**
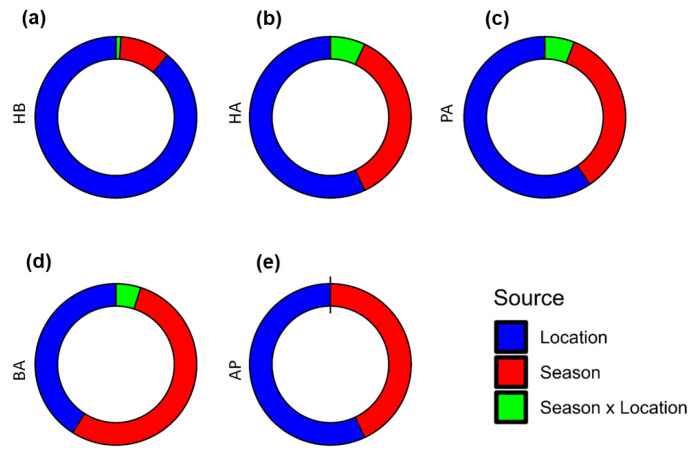
Effect sizes of main and interaction effects of parameters on different characters. The figure shows the relative importance of the main (season and location) effects and interaction (season × location) effects of the factors on five characters: (**a**) hygienic behavior, (**b**) honey area, (**c**) pollen area, (**d**) brood area, and (**e**) adult population, using doughnut diagrams.

**Figure 6:**
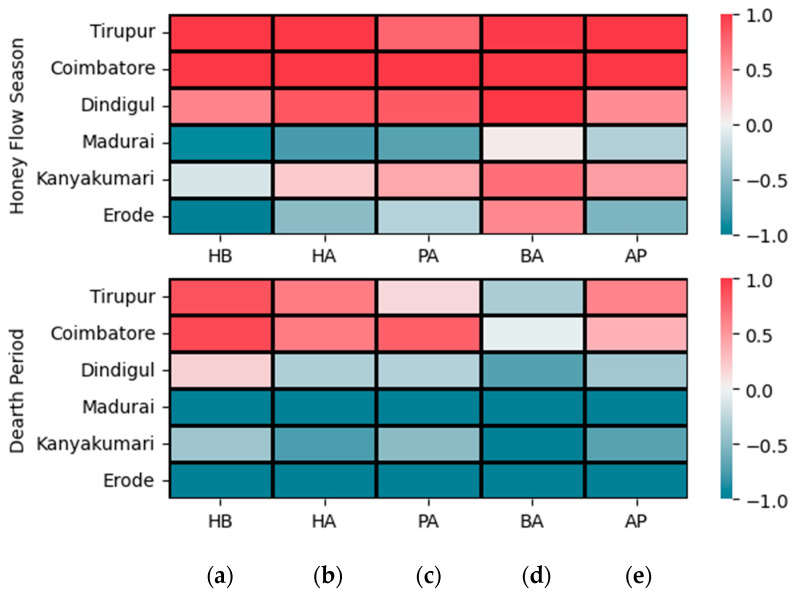
Degree of performance of honey bee colonies. Cohen’s d statistic computed for pairwise mean comparison was summed across interactions and averaged to study the performance of colonies across six locations (Tirupur, Coimbatore, Dindigul, Madurai, Kanyakumari, and Erode) and two seasons (honey flow season and dearth period) measured by five different characters, (**a**) hygienic behavior, (**b**) honey area, (**c**) pollen area, (**d**) brood area, and (**e**) adult population, using a heat map. The color gradient from red to blue indicates the range of effect sizes from 1.0 to −1.0. The red color gradient indicates higher effect sizes, while the blue color gradient depicts the least effect sizes. White color represents non-significant effect sizes.

The results of the Aligned Rank Transformation (ART)-ANOVA for repeated measures are presented in Table 1. Furthermore, the pairwise mean comparison among characters measured from different locations and during distinct periods, the relative importance of main effects (Season and Location) and interaction effects, and the degree of performance of honey bee colonies are presented in Figure 4, Figure 5, and Figure 6, respectively.

#### 3.4.1. Hygienic Behavior

The ART-ANOVA for repeated measures results revealed significant effects of both season (*p* < 0.001, $\eta_{p}^{2}$ = 0.07) and location (*p* < 0.001, $\eta_{p}^{2}$ = 0.68) on the honey bee colonies’ hygienic behavior (HB), with moderate and very high effect sizes, respectively (Table 1 and Figure 4a, Figure 5a and Figure 6a). The variability among locations surpassed that among seasons. No interaction between season and location was observed, indicating a consistent season effect across locations. Colonies exhibited significantly better performance during the honey flow season, regardless of their location. Post-hoc pairwise mean comparisons identified Coimbatore (d = 2.28) as having the highest HB, followed by Tirupur (d = 2.07). Colonies in Madurai (d = −2.19) and Erode (d = −2.39) exhibited relatively poor HB. Ranking locations based on colony HB from best to least are as follows: Coimbatore, Tirupur, Dindigul, Kanyakumari, Madurai, and Erode. Notably, variation was lower among colonies with superior HB compared to those with poor HB.

#### 3.4.2. Honey Area

Regarding the honey area (HA) of bee colonies, both season (*p* < 0.001) and location (*p* < 0.001) significantly explained a substantial amount of variation with a very high effect size (Table 1 and Figure 4b, Figure 5b and Figure 6b). Notably, the variation explained by the location parameter ($\eta_{p}^{2}\;$= 0.65) slightly exceeded that explained by seasons ($\eta_{p}^{2}$ = 0.42). In contrast to hygienic behavior (HB), there was a significant interaction effect of the factors on HA with a high effect size (*p* < 0.001, $\eta_{p}^{2}$ = 0.08). Colonies with the best HA were in Coimbatore (d = 1.49), followed by Tirupur (d = 1.44), both during the on-season as indicated by the average Cohen’s d statistic across all pairwise mean comparisons. Additionally, it was observed that the HA of colonies from these districts relatively declined and equated during the dearth period (d = 0.68). The colonies in Dindigul exhibited the next best honey area during the honey flow season (d = 0.87), though a considerable decrease was noted during the off-season (d = −0.33). Colonies from Kanyakumari had a lower honey area compared to other districts during the honey flow season (d = 0.21), which further declined during the dearth period (d = −0.78). Erode (d = −0.98) and Madurai (d = −1.15) districts had the poorest honey area in both seasons.

#### 3.4.3. Pollen Area

Examining the pollen area (PA) of honey bee colonies revealed the substantial impact of both season (*p* < 0.001) and location (*p* < 0.001) parameters (Table 1 and Figure 4c, Figure 5c and Figure 6c). Consistent with observations in HB and HA, the influence of location ($\eta_{p}^{2}$ = 0.52) was more pronounced than that of season ($\eta_{p}^{2}$ = 0.30). A discernible interaction effect between factors was identified (*p* < 0.05), although not as prominent as seen in HA. Notably, colonies in Coimbatore exhibited the highest PA during the on-season, demonstrating a significant deviation (d = 1.57) from colonies in other locations. Dindigul colonies showcased a commendable PA during the on-season (d = 0.85), comparable to Coimbatore during the dearth period (d = 0.84). The colonies in Tirupur displayed a noteworthy PA during the honey flow season (d = 0.80), marginally lower than Dindigul during the on-season. Kanyakumari’s colonies experienced a slight improvement in the PA during the honey flow season (d = 0.41) but suffered a substantial decline during the off-season (d = −0.52). In a departure from earlier instances, the colonies in Tirupur exhibited a lower PA during the off-season (d = 0.15) compared to Kanyakumari during the honey flow season. Conforming to the prevailing pattern, Erode (d = −0.83) and Madurai (d = −1.07) exhibited a subpar PA compared to all other locations.

#### 3.4.4. Brood Area

The brood area (BA) of honey bee colonies exhibited significant variations influenced by both season (*p* < 0.001) and location (*p* < 0.001), as evident from the results (Table 1 and Figure 4d, Figure 5d and Figure 6d). In contrast to earlier discussions, the seasonal impact ($\eta_{p}^{2}$ = 0.72) surpassed that of location ($\eta_{p}^{2}$ = 0.05). Interaction effects ($\eta_{p}^{2}$ = 0.07) were also notable but of moderate effect size. Irrespective of location, colonies consistently demonstrated a significantly higher BA during the on-season compared to the dearth period. Notably, during the honey flow season, Coimbatore’s colonies (d = 1.64) displayed the most substantial BA, followed by Dindigul (d = 1.26) and Tirupur (d = 1.04). A moderate BA was observed in Kanyakumari’s (d = 0.72) and Erode’s (d = 0.60) colonies. Madurai’s colonies recorded the lowest BA during the honey flow season (d = 0.05). In the off-season, the BA significantly decreased across all locations, with colonies of Erode (d = −1.54), Madurai (d = −1.41), and Kanyakumari (d = −1.17) displaying the lowest BA, significantly differing from other locations. The colonies in Coimbatore (d = −0.06) relatively maintained the highest BA, followed by Tirupur (d = −0.37) and Dindigul (d = −0.76), with Tirupur outperforming Dindigul during the dearth period.

#### 3.4.5. Adult Population

The adult population (AP) in honey bee colonies was significantly influenced by both the season (*p* < 0.001, $\eta_{p}^{2}$ = 0.37) and location (*p* < 0.001, $\eta_{p}^{2}$ = 0.49) parameters, indicating a substantial effect size (Table 1 and Figure 4e, Figure 5e and Figure 6e). However, the impact of location on the AP was relatively lower compared to other characteristics. There was no evidence supporting interaction effects between factors on the AP $\left(p=0.9941,\;\eta_{p}^{2}=0.00\right)$. Similar to HB, honey bee colonies exhibited better performance during the honey flow season period than the dearth period, regardless of their locations. The Tirupur district colonies (d = 0.98) displayed the highest adult population, significantly differing from all other locations, followed by Coimbatore (d = 0.75) with a slightly lower effect size. Dindigul colonies (d = 0.09) ranked third in terms of adult population, albeit with a small effect size. Colonies in Kanyakumari (d = −0.12), Madurai (d = −0.75), and Erode (d = −0.95) districts exhibited a smaller AP, with Erode and Madurai having relatively very small effect sizes.

### 3.5. Colony Selection Based on the Selection Index

The selection index was calculated from the summation of Z scores of traits for all the colonies (n = 117). The results of the selection index of colonies with a top 10 rank are provided in Table 2, and detailed results of the selection index are provided in Table S5. The results showed that colony T4 (from Tirupur) had the highest selection index (107.6779), ranking first among all the colonies, followed by C2 and C7 (from Coimbatore). It is noteworthy that colony T4 was observed with the highest Z score in the HA parameter but not in the other parameters. However, it fell in the range of the top five rankings in other parameters (2nd in AP, 3rd in BA, and 5th in HB and PA).

### 3.6. Hierarchical Agglomerative Clustering (HAC)

**Figure 7:**
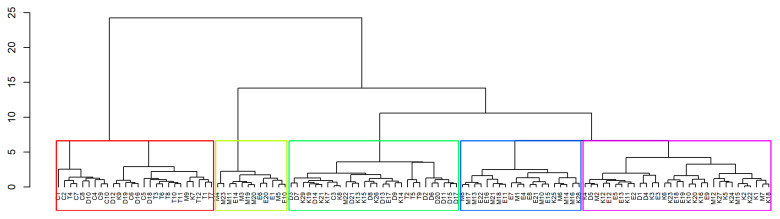
Hierarchical agglomerative clustering dendrogram for *A. cerana indica* colonies Note: In the Colony ID, the alphabet letters indicate the location (C—Coimbatore, D—Dindigul, E—Erode, K—Kanyakumari, M—Madurai, and T—Tirupur) and numbers represent corresponding colony number. For example, T4 is the colony no. 4 in Tirupur. The different colors represent different clusters (red—Cluster I, yellow—Cluster II, green—Cluster III, blue—Cluster IV, purple—Cluster V).

Cluster analysis, depicted in Figure 7, was conducted based on similarity coefficients among pairs of colonies, resulting in the categorization of 117 colonies into Cluster I (n = 26), Cluster II (n = 28), Cluster III (n = 31), Cluster IV (n = 19), and Cluster V (n = 13). Cluster I, comprising 26 colonies (nine colonies each from Tirupur and Coimbatore, five from Dindigul, two from Kanyakumari, and one colony from Madurai), displayed the highest mean value for hygienic behavior (HB) at 90.4 ± 2.77%, making these colonies prime candidates for breeding programs aimed at enhancing hygienic behavior and colony performance. Notably, Cluster I stood separate from other clusters, showcasing superior performance over others. The clusters II, III, IV, and V demonstrated moderate to low performance, requiring targeted improvements through selective breeding. These findings underscore the substantial diversity among the colonies utilized in the study. This suggests that colonies from Cluster I hold potential for further screening and maintaining breeding lines, while the diversity observed across clusters emphasizes the varied characteristics present in the studied honey bee colonies.

## 4. Discussion

We conducted field experiments to observe and screen colonies for better performance with special emphasis on hygienic behavior in six different districts of Tamil Nadu, India, and two seasons. The six study locations represent distinct genetic lineages of *Apis cerana indica*, as the beekeepers practice stationary beekeeping, minimizing the likelihood of genetic mix-up. Comparing the mean values of key metrics between honey flow season and the dearth period provides valuable insights into the nuances of honey bee colony dynamics. The purpose of studying HB in the honey flow and dearth period is that the colony growth parameters are expected to be high in the honey flow period, and TSBV is a disease that manifests more during higher brood development [36].

Honey flow season revealed marginally elevated mean values across crucial parameters, including honey bee population, honey area, pollen area, and brood area, indicative of heightened hive activity during this timeframe. Honey flow season consistently demonstrates a slightly higher variability in colony growth parameters compared to the dearth period.

Our findings reveal significant variability among the compared colonies in terms of hygienic behavior and other traits related to colony growth parameters. The predominant influencing factor was identified as the location, encompassing the aggregate impact of both abiotic and biotic elements within a specific environment [37]. The duration of the active season, coupled with food accessibility, played a substantial role in shaping the developmental trajectories of colonies across diverse locations [38]. This impact likely extended beyond mere colony development, potentially influencing overall colony performance. As outlined by previous studies, colonies across all locations adhered to a standardized protocol encompassing mandatory procedures, assessment timing, and sampling methods [33]. While these fundamental activities were universally implemented, the colonies were additionally managed following the beekeeping practices prevalent in each local context, thereby augmenting the influence associated with geographical location.

Honey bees encountering fluctuations in weather undergo stress, which can affect multiple facets of their physiology and behavior [39]. Variations in the manifestation of hygienic behavior across seasons have been frequently documented [40,41], although conflicting reports also exist [42]. The interplay between season and location likely contributes to unique combinations of floral availability and nectar flow, well-known factors influencing hygienic behavior expression [19,43,44,45]. Hygienic behavior in honey bee colonies varies significantly among and within populations and subspecies, influenced by habitat, genetic lineage, and geographic differences [46,47,48,49]. In our case, there was only a minimum effect caused by season and no interaction between the season and the location, considering the hygienic behavior trait. Hygienic behavior in honey bee colonies is stable throughout the season and largely unaffected by environmental factors or population size, allowing for consistent screening regardless of nectar availability or brood levels [42,50]. However, there was a significant correlation between colony growth parameters and hygienic behavior. Several studies indicate a positive correlation between hygienic behavior and honey production, suggesting that selecting for increased hygienic behavior may also enhance honey yields, though this correlation warrants further investigation in larger populations [6,50,51,52]. A previous study on colony strength found that transferring hygienic and non-hygienic colonies from 10-frame field hives to 2-frame observation hives significantly reduced the hygienic response in hygienic bees but had no effect on the response of non-hygienic bees [53].

The Thai sacbrood virus has decimated the majority of *A. cerana indica* colonies, leaving only those that survived in the wild to repopulate hives in India. In this context, while sacbrood virus particles are harmless to adult bees, these bees act as asymptomatic carriers [54]. Consequently, there is always a low-level disease presence with minimal symptomatic expression in larvae due to the lower viral particle titer. It is unclear whether the surviving colonies possess genetic resistance, exhibit hygienic behavior, or simply benefit from isolation from infected colonies. Conceptually, early detection and rapid response by workers, which prompt the timely removal of infected brood, can effectively reduce pathogen spread within colonies [18]. We hypothesize that colonies capable of sensing physiological changes in broods with lower viral titers can efficiently perform hygienic behavior, thereby preventing the spread and establishment of the disease and maintaining brood size. This may explain the positive correlation between colony size and hygienic behavior activity. Although further evidence is needed, this study opens avenues for future research.

The analysis of various parameters, including honey area (HA), pollen area (PA), brood area (BA), and adult population (AP), provided valuable insights into the dynamics of honey bee colonies across different seasons, specifically the honey flow season and the dearth period, and locations. For HA, both season and location played significant roles, with the latter exerting a slightly stronger influence [55]. The availability of pollen depends not only on the diversity of the landscape but also on seasonal variations [56]. Similarly, our PA data demonstrated significant fluctuations, with location exerting a more pronounced influence than seasonal variations. Notably, in Coimbatore, where coconut plantations ensure a year-round supply of pollen, the PA in colonies consistently maintained high scores even during the dearth period. This underscores the influential role of coconut plantations in sustaining robust pollen availability in the region. Intriguingly, the BA displayed notable seasonal variations, with colonies generally exhibiting a higher BA during the honey flow season. Coimbatore colonies consistently showcased the most substantial brood area, emphasizing the influence of both season and location. The AP was significantly influenced by both season and location, with Tirupur colonies consistently maintaining the highest adult population during the honey flow season, underscoring the interconnected effects of these factors on colony performance. Overall, these findings highlight the importance of considering both seasonality and location-specific factors in understanding the relation between the honey bee colony traits effectively.

In breeding programs, selecting a colony for a new trait should not undermine progress on previously selected traits, as trade-offs, such as improved hygienic behavior potentially leading to lower honey yields or increased swarming and defensive behavior, can represent significant costs for breeders [6]. To ward off these factors, they are selected for multiple traits along with hygienic behavior. In the present study, the colonies with superior hygienic behavior and other colony parameters were identified using the selection index (SI) calculation and HAC analysis. Intriguingly, the colonies in Cluster I of the HAC analysis depicted the top 26 colonies from the SI results as well (irrespective of the rank order) (Table S5). These colonies can be used for further selection processes, genetic parameter analysis, and maintenance of breeding lines to develop future breeding programs.

## 5. Conclusions

In conclusion, our field experiments, conducted in six districts of Tamil Nadu, India, across two seasons, focused on the performance of *A. cerana indica* colonies with an emphasis on hygienic behavior. The honey flow season exhibited marginally elevated mean values across key colony metrics, suggesting heightened hive activity during this period. Our findings revealed significant variability in hygienic behavior among different locations. Hygienic behavior showed a stable expression across seasons and was largely unaffected by environmental factors, suggesting consistent screening potential. Despite minimal effects caused by the season and no interaction between season and location in terms of hygienic behavior, a significant correlation was observed between hygienic behavior and colony growth parameters, such as honey area, pollen area, brood area, and adult population. However, further investigation with larger populations is warranted. Our comprehensive analysis of colony selection, including the selection index calculation and hierarchical agglomerative clustering analysis, identified superior colonies for future breeding programs, highlighting the importance of selecting multiple traits to avoid trade-offs. These findings underscore the necessity of considering both seasonal and location-specific factors in breeding programs to enhance overall colony performance and ensure sustainable beekeeping practices.

## Figures and Tables

**Table 1:** Aligned Rank Transformation (ART)-Analysis of Variance for repeated measures.

Source	Df	Residual	HB		HA		PA		BA		AP	
			F Value	$\eta_{p}^{2}$	F Value	$\eta_{p}^{2}$	F Value	$\eta_{p}^{2}$	F Value	$\eta_{p}^{2}$	F Value	$\eta_{p}^{2}$
Season	1	222	17.61	0.07	157.92	0.42	94.14	0.30	564.21	0.72	131.86	0.37
Location	5	222	93.61	0.68	82.17	0.65	47.39	0.52	51.78	0.54	43.25	0.49
Season × Location	5	222	0.28	0.01	4.02	0.08	2.23	0.05	3.42	0.07	0.09	0.00

**Table 2:** Selection index of colonies with a top 10 rank.

Colony ID	Selection Index	Rank
T4	107.6779	1
C2	104.3092	2
C7	99.05145	3
C9	98.61222	4
C10	92.61824	5
C4	91.99659	6
D10	91.68568	7
C8	81.35574	8
T6	71.77229	9
T12	71.70287	10

## Data Availability

Data will be made available on request.

## Supplementary Materials

The following supporting information can be downloaded at: https://www.mdpi.com/article/10.3390/insects15080598/s1, Table S1. Details on locations studied. Table S2. Descriptive statistics of observed colony traits in different seasons. Table S3. Descriptive statistics of observed colony traits in different locations. Table S4. Correlation analysis of different parameters in different locations. Table S5. Selection index of colonies.
